# Is Oral Microflora Related to Development of Malfunction in Patients Using Voice Prosthesis?

**DOI:** 10.3390/jcm13123492

**Published:** 2024-06-14

**Authors:** Angelo Immordino, Francesco Dispenza, Federico Sireci, Riccardo Anzalone, Palmira Immordino, Cinzia Calà, Salvatore Gallina, Francesco Lorusso

**Affiliations:** 1 Otorhinolaryngology Section, Department of Biomedicine, Neuroscience and Advanced Diagnostics (BiND), University of Palermo, 90127 Palermo, Italy; angelo.immordino182@gmail.com (A.I.); francesco.dispenza@gmail.com (F.D.); riccardo.anzalone01@you.unipa.it (R.A.); salvatore.gallina@unipa.it (S.G.); 2 Otorhinolaryngology Section, Department Precision Medicine in Medical, Surgical and Critical Care (Me.Pre.C.C.), University of Palermo, 90127 Palermo, Italy; federico.sireci@unipa.it; 3 Hygiene and Preventive Medicine Section, Department of Health Promotion, Maternal and Infant Care, Internal Medicine, and Medical Specialties (PROMISE), University of Palermo, 90127 Palermo, Italy; palmira.immordino@gmail.com; 4 Microbiology and Virology Complex Operative Unit, University Hospital “P. Giaccone”, 90127 Palermo, Italy; cinzia.cala@unipa.it

**Keywords:** laryngectomy, voice prostheses, microorganism, infection

## Abstract

**Background**: this prospective study investigated the correlation between the oral bacterial microflora and the microflora found in voice prostheses (VPs) among 20 patients who had undergone laryngectomy. The aim was to explore the associations between the microflora’s presence and the malfunction of VPs, along with the association between the predominant microorganism and the longevity of VPs. **Methods**: the research process included gathering medical histories, conducting ENT examinations, replacing VPs, and performing check-ups every four months for a period of 15.5 months. Additionally, microbiological examinations, blood tests, and voice change surveys were conducted. **Results**: a correlation between the microflora isolated from VPs and that from oral rinses was demonstrated in a large percentage of patients who experienced a loss of prosthetic functional efficiency. The correlation analysis between the type of microorganism and the lifespan of VPs showed a non-significant Pearson correlation coefficient (r = 0.043, *p* = 0.678). **Conclusions**: there is no significant linear correlation between the predominant microorganism and the average lifespan of VPs.

## 1. Introduction

For 15 to 40% of patients with locally advanced or recurrent cancer of the larynx or hypopharynx, the definitive treatment modality is total laryngectomy (TL) or laryngopharyngectomy, either as a primary or salvage surgery. Surgical resection leads to the loss of the natural laryngeal speaking voice and impacts nasal function and airway control and may result in dysphagia, significantly affecting the quality of life (QOL) among survivors [1]. Moreover, speech, a fundamental aspect of human communication, becomes a critical challenge after laryngectomy, making voice restoration a paramount yet difficult goal for surgeons and speech therapists. Since the successful performance of the first laryngectomy for laryngeal cancer by Billroth in 1873, numerous attempts have been devoted to voice rehabilitation for these patients. In 1874, Gussenbauer proposed the use of an artificial larynx to reroute air from the trachea to the pharynx. Since then, voice rehabilitation after TL has evolved considerably, incorporating esophageal speech, electronic devices for sound generation, and tracheoesophageal puncture (TEP) accompanied by voice prosthesis (VP) placement.

While the process of acquiring esophageal speech shows lower speech quality and functional effectiveness compared to other methods, it presents advantages in terms of cost-effectiveness and reduced maintenance requirements. Conversely, the utilization of an electrolarynx is considered to be less favored due to its limitations in achieving optimal speech clarity and natural voice quality [2].

A VP is a unidirectional silicone rubber prosthetic valve that connects the trachea and upper esophagus. Since its advent, it has been considered the gold standard in voice rehabilitation after laryngectomy, providing speech powered by the lungs and, therefore, a physiologically better and significantly clearer voice [3].

Several studies have compared the outcomes achieved through esophageal speech rehabilitation and VP rehabilitation by assessing different questionnaires, such as the Voice Handicap Index (VHI) and the Voice-Related Quality of Life (V-RQOL), demonstrating that VPs allow for higher scores on both questionnaires [4,5,6,7,8,9,10].

VPs are temporary implants with a limited lifespan. The most common reason for the replacement of these devices is the leakage of fluids through the VP, which typically indicates the inadequate closure of the one-way valve mechanism [11]. This malfunction is primarily due to the development of a biofilm on the valve, consisting of bacteria and Candida species, hindering its proper closure [12]. Despite their critical role, the persistent development of biofilms on these prostheses poses an ongoing challenge. Biofilms are complex microbial communities encased in a self-generated extracellular matrix that promotes the adhesion and protection of microorganisms. Anaerobic and microaerophilic pathogens, particularly adept at surviving in low-oxygen environments, contribute significantly to the initiation and persistence of biofilms on VPs [13]. The objective of this prospective study is to explore the associations between the microflora’s presence and the malfunction of VPs, along with the association between the predominant microorganism and the longevity of VPs.

## 2. Materials and Methods

Between 2020 and 2023, at the ENT department of the University Hospital “Paolo Giaccone” in Palermo, Italy, we conducted a prospective study focusing on patients who had previously undergone total laryngectomy for squamous cell carcinoma of the larynx and were using VPs. These patients had reported issues such as fluid leakage from or around the inner valve of the VP, difficult or impossible phonation, and the worsening of the voice quality.

The inclusion criteria encompassed patients over 18 years of age, patients undergoing total laryngectomy, patients with voice prostheses (with primary technique), and patients using HME. The exclusion criteria encompassed patients unable to understand and answer the questionnaires; patients with cognitive function impairments; patients who underwent associated radiotherapy, chemotherapy, or flap reconstruction; and patients suffering from gastroesophageal reflux. These latter factors are already known to decrease the average lifespan of VPs [14]. As part of the evaluation process, each patient provided their medical history and underwent a comprehensive ear, nose, and throat examination, including a fibroscopic assessment of the upper aerodigestive tract and a tracheoscopic evaluation. Following these evaluations, the patients received a VP replacement and were scheduled for follow-up visits every four months for a year at our department. They were also encouraged to arrange an earlier check-up if they experienced any complications, like difficulties in speaking, coughing during meals, leakage from the voice prosthesis, or the dislocation of the device. For purpose of this study, we selected three types of indwelling silicone rubber VPs that are widely used internationally and suitable for nearly all laryngectomy patients (Table 1).

During the follow-up period, the patients were subjected to a microbiological examination through VP brushing to assess the microflora and oral rinse samples to determine the oral commensal flora. These samples were forwarded to the microbiology department of our university hospital for microbiological and cultural examination.

For the isolation, cultivation, and identification of aerobes, anaerobes, and fungi, VP brushings from the inner parts of the prostheses and oral rinses from the twenty patients were sent to the microbiology and virology laboratory. After resuspending the VP brushings in saline solution (0.9% NaCl), both types of samples were centrifuged at 20,000× *g* for 10 min at 4 °C, and the pellet was inoculated into different growth media for fastidious and non-fastidious microorganisms. Colonies grown after overnight incubation at 37 °C were identified using MALDI-TOF mass spectrometry (Bruker Biotyper). Additionally, for microorganisms such as *Staphylococcus aureus*, *Pseudomonas aeruginosa*, and *Enterobacterales*, the antimicrobial susceptibility was assessed using the BD Phoenix automated system. Additionally, a blood test with a leukocyte count was conducted to assess the presence of any systemic infections that might influence the microbiological activity at the VP site. Moreover, voice change questionnaires such as the Voice Handicap Index (VHI) and the Voice-Related Quality of Life (V-RQOL) were administered to the patients to evaluate whether inner valve fluid leakage was connected to the vocal ineffectiveness of the prosthesis [15]. These instruments are commonly used to assess dysphonia and its effects on patients’ lives.

The V-RQOL, specifically, is a self-administered, 10-item assessment tool designed to capture the unique challenges of voice disorders. It was developed to standardize post-treatment voice-related changes by measuring two primary constructs: physical and social–emotional functioning [16]. Participants rate ten statements on a scale from 1 (indicating no problem) to 5 (indicating a severe problem), with the scores for each domain reflecting the degree to which voice impairments impact their everyday vocal communication [17]. These scores were derived using a standard scoring algorithm, and the questions were ranked by their mean values to highlight the most significant issues for this patient group.

All data, including information on the oral microflora, the VP characteristics, the recurrence of complications at the VP site, and the average lifespans of the different VPs used, were meticulously collected and analyzed.

### Statistical Analysis

The statistical analysis of the data was conducted using the JASP (Jeffreys’s Amazing Statistics Program) software, version 0.18.1. After data preparation, a correlation analysis was performed to evaluate the relationship between the predominant type of microorganism and the average lifespan of the VPs. Specifically, the Pearson correlation coefficient was used to assess the presence of a linear correlation between the variables under examination. A *p*-value < 0.05 was considered significant.

## 3. Results

### 3.1. Study Group

Twenty patients were recruited at our department for this prospective study, including 17 males and 3 females. The mean age was 63.7 (±8.6) years.

The distribution of the prostheses in the study group is shown in Table 2.

All patients had the VP implanted concurrently with their laryngectomy. The follow-up period averaged 465 (±81.7) days. During the annual follow-up, 19 patients experienced two episodes of leakage, while one patient encountered a leakage three times. Patients reported the onset of leakage, on average, at 135 (±32.7) days following the initial implantation. Eighteen reported a leakage within the valve, while two experienced leakages from the perivalvular region (both using the Blom-Singer Dual Valve 20 Fr—8 mm). The yearly average for voice prosthesis replacements post-laryngectomy was 2.05 times. Twelve patients underwent replacement with the same type of VP, while eight received a VP of a different model or diameter.

**Figure 1:**
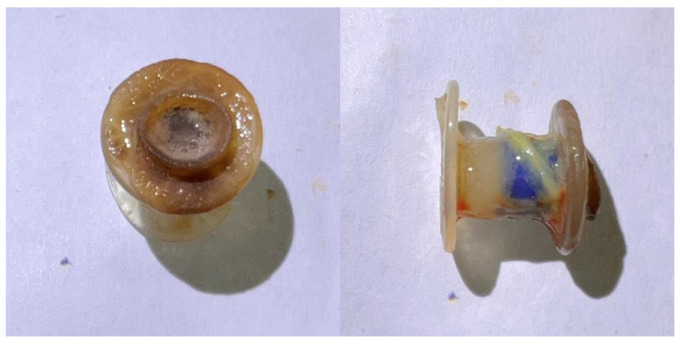
Macroscopic views of a colonized VP.

No significant variations were found in the laboratory tests, especially concerning the leukocyte count. Figure 1 shows macroscopic views of a colonized VP.

### 3.2. Questionnaires

Questionnaires assessing the phonatory efficiency of the prostheses were administered following the first report of partial ineffectiveness and the subsequent discovery of valve fluid leakage. According to the Voice Handicap Index (VHI), 30% of the patients (six out of twenty) had a minimal handicap (score 0–30), 50% (ten patients) had a moderate handicap (score 31–60), and 20% (four patients) had a severe handicap (score over 61).

The Voice-Related Quality of Life (V-RQOL) questionnaire highlighted that the issue described in question 1 (“Because of my voice, I have trouble speaking loudly or being heard in noisy situations”) posed the greatest challenge, whereas that in question 8 (“Because of my voice, I avoid going out socially”) was the least problematic. This observation aligns with existing scoring procedures, suggesting that scores of five indicate the greatest impact on quality of life (QOL), whereas scores of one indicate a minimal impact [16].

### 3.3. Correlation between VP Brushing and Oral Rinse Microflora

**Figure 2:**
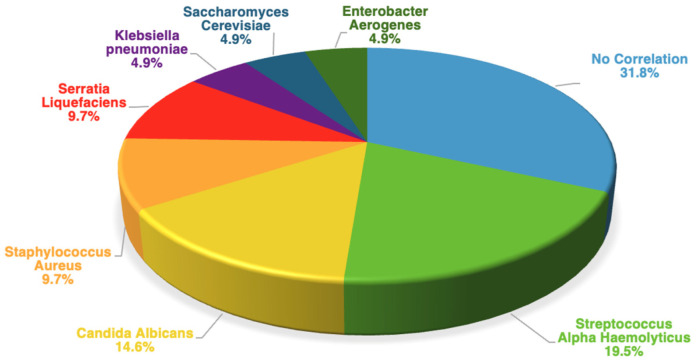
Correlation between VP brushing and oral rinse microflora.

Of the 19 patients who experienced a VP leakage twice during the follow-up, we collected a total of 38 pairs of samples (38 from oral rinses and 38 from VP brushings). From the single patient who experienced a VP leakage three times, we collected three pairs of samples. We therefore analyzed a total of 41 pairs of samples obtained from the VP brushings and oral rinses and observed a positive correlation between the two microbiological samples in 68.2% of the cases (28/41): 19.5% with *Streptococcus alpha-haemolyticus* (8/41); 14.6% with *Candida albicans* (6/41); 9.7% with *Staphylococcus aureus* (4/41); 9.7% with *Serratia liquefaciens* (4/41); 4.9% with *Klebsiella pneumoniae* (2/41); 4.9% with *Enterobacter aerogenes* (2/41); and 4.9% with *Saccharomyces cerevisiae* (2/41) (Figure 2).

### 3.4. Isolated Microflora in VP Brushings

**Figure 3:**
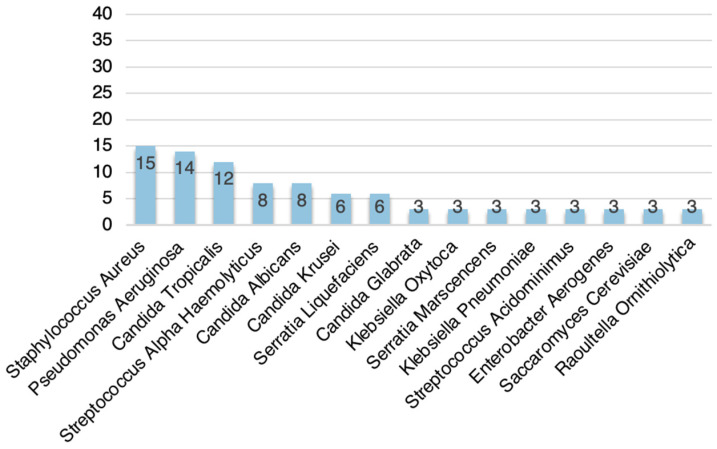
Isolated microflora in VP brushings.

In the 41 samples of VP brushings, a total of 15 different microorganisms were isolated. In all VP brushing samples, the isolated flora was heterogeneous and showed co-colonization by different microorganisms. The isolated microorganisms were *Staphylococcus aureus* in 36.6% of the samples (15/41), *Pseudomonas aeruginosa* in 34.1% (14/41), *Candida tropicalis* in 29.3% (12/41), *Streptococcus alpha-haemolyticus* in 19.5% (8/41), *Candida albicans* in 19.5% (8/41), *Candida krusei* in 14.6% (6/41), *Serratia liquefaciens* in 14.6% (6/41), *Candida glabrata* in 7.3% (3/41), *Klebsiella oxytoca* in 7.3% (3/41), *Serratia marscencens* in 7.3% (3/41), *Klebsiella pneumoniae* in 7.3% (3/41), *Streptococcus acidominimus* in 7.3% (3/41), *Enterobacter aerogenes* in 7.3% (3/41), *Saccaromyces cerevisiae* in 7.3% (3/41), and *Raoultella ornithiolytica* in 7.3% (3/41) (Figure 3).

### 3.5. Relationship between Predominant Microorganism and VP Lifespan

The analysis of the average lifespan of the VPs, in relation to the predominant microorganisms, was conducted on a sample of 41 VP brushings. The predominant microorganisms were identified in the samples, including *Staphylococcus aureus* (n = 11), *Candida tropicalis* (n = 9), *Pseudomonas aeruginosa* (n = 7), *Streptococcus alpha haemolyticus* (n = 5), *Candida albicans* (n = 5), and *Candida krusei* (n = 4).

The correlation analysis between the type of microorganism and the lifespan of the VPs showed a non-significant Pearson correlation coefficient (r = 0.043, *p* = 0.678). This suggests that there is no significant linear correlation between the predominant microorganism and the average lifespan of VPs.

## 4. Discussion

The main objective of our study was to analyze whether there was a correlation between the pathogens present in the oral rinses and those residing in the biofilm of the vocal prosthesis.

Based on the results of our analysis, where we examined 41 pairs of samples obtained from VP brushings and oral rinses, several key findings emerged. Firstly, we observed a positive correlation between the microbiological compositions of these two types of samples in a significant portion of cases, specifically in 68.2% of the samples (28 out of 41). This notable overlap in the microbial populations suggests a possible mechanism of descending colonization from the oral cavity, leading to VP dysfunction.

Among the predominant microorganisms identified in these samples, *Streptococcus alpha-haemolyticus*, *Candida albicans*, *Staphylococcus aureus*, *Serratia liquefaciens*, *Klebsiella pneumoniae*, *Enterobacter aerogenes*, and *Saccharomyces cerevisiae* were the most prevalent. Notably, *Streptococcus alpha-haemolyticus* and *Candida albicans* were the most frequently detected, accounting for 19.5% and 14.6% of the samples, respectively.

This observation highlights the potential role of these microorganisms in VP colonization and the development of associated malfunctions. *Streptococcus alpha-haemolyticus* is a common inhabitant of the oral cavity and has been implicated in various infections, including those affecting the upper respiratory tract. Similarly, *Candida albicans* is a known opportunistic pathogen that can cause oral candidiasis and has been associated with VP-related complications such as fungal biofilm formation.

Furthermore, the presence of other microorganisms, such as *Staphylococcus aureus*, *Serratia liquefaciens*, *Klebsiella pneumoniae*, *Enterobacter aerogenes*, and *Saccharomyces cerevisiae*, underscores the diverse nature of the microbial community inhabiting the VP and its surrounding environment. These findings suggest that multiple microbial species may contribute to the colonization of VP surfaces, potentially leading to biofilm formation, material degradation, and functional impairment.

Among the bacterial species isolated from the VPs, *Staphylococcus aureus* and *Pseudomonas aeruginosa* were the most prevalent, detected in 36.6% and 34.1%, respectively. These findings are consistent with previous studies highlighting the prominence of these microorganisms in VP-related infections and malfunctions [11,18]. The main yeast strains isolated from the biofilms on dysfunctional VPs were *Candida tropicalis* and *Candida albicans.* The results regarding the types of yeast most frequently isolated from the VPs are consistent with the findings from other studies [19,20]. However, in our case series, the species most frequently isolated was not *Candida albicans* but *Candida tropicalis*, occurring in 29.3% of cases.

The adherence of *Candida* species hyphae plays a crucial role in initiating biofilm formation: it can form a three-dimensional network on the surfaces of VPs, contributing to prosthesis malfunction, by expressing surface adhesion molecules Eap1 and Als3 within the biofilm structure. These molecules act as specific surface receptors responsible for binding bacteria, including *Viridans* group streptococci and *Staphylococcus aureus* [21,22,23,24]. The penetration of yeast filaments is facilitated by the enzymatic degradation of silicone, and its degradation might provide nutrients for growing yeasts [21,25]. *Staphylococcus aureus* emerged as the most frequently isolated bacterial species in the VPs, aligning with what is reported in the literature [11,18].

The presence of such a diverse array of microorganisms underscores the importance of comprehensive microbial surveillance and targeted interventions to mitigate the risk of VP-related infections and complications. Understanding the specific microbial profiles associated with VP colonization can inform strategies for antimicrobial prophylaxis, biofilm prevention, and therapeutic interventions aimed at optimizing VP function and prolonging the device’s lifespan.

Regarding the correlation between the predominant microorganism and the VP’s lifespan, the results suggest that there are no statistically significant relationships. No species, whether yeasts or bacteria, exhibited a statistically significant influence on the lifespan of the VPs. This may indicate that the causes of material disintegration in prostheses are multifactorial, beyond the dominance of specific pathogens. Furthermore, it should be noted that obtaining an exact estimate of the lifespan of a prosthesis was challenging because the patients were brought to our attention only when the vocal defect was perceived, and the leakage of liquids was not minimal.

The administration of questionnaires assessing the phonatory efficiency of the prostheses provided essential insights into the impact of valve fluid leakage on patients’ vocal functionality and quality of life. Following the initial report of partial ineffectiveness and the subsequent discovery of the valve fluid leakage, the VHI revealed that 30% of the patients (six out of twenty) experienced a minimal handicap (score 0–30), 50% (ten patients) had a moderate handicap (score 31–60), and 20% (four patients) had a severe handicap (score over 61). These findings suggest that while a significant proportion of the patients encountered moderate to severe challenges, a notable segment experienced minimal impairment.

The V-RQOL questionnaire further highlighted specific challenges faced by the patients. The issue described in question 1 (“Because of my voice, I have trouble speaking loudly or being heard in noisy situations”) posed the greatest challenge, indicating that the inefficiency of the prostheses significantly affected the patients’ ability to communicate effectively in noisy environments. Conversely, that described in question 8 (“Because of my voice, I avoid going out socially”) was the least problematic, suggesting that social avoidance due to vocal issues was less prevalent. This observation aligns with the existing literature, which suggests that higher scores (5) indicate the greatest impact on QOL, whereas lower scores (1) indicate a minimal impact.

These findings are consistent with previous studies highlighting the importance of vocal efficiency and its direct correlation with patients’ overall QOL. Vocal inefficiency, particularly in challenging acoustic environments, can significantly impair social interactions and daily communication. The disparity between the impact on vocal loudness and social activities underscores the multifaceted nature of V-RQOL, where some aspects of vocal impairment are more tolerable or adaptable than others. For instance, while patients might struggle to be heard in noisy settings, this does not necessarily translate to broader social withdrawal, indicating a complex interplay between the different dimensions of vocal impairment and social behavior [2,5,7,9,10].

The results from our study underscore the significant burden that valve fluid leakage places on phonatory efficiency and QOL among patients with vocal prostheses. Addressing this issue requires a comprehensive approach that not only targets the mechanical and functional aspects of the prostheses but also considers the broader psychosocial implications.

## 5. Conclusions

In conclusion, our prospective study aimed to investigate the correlation between the oral bacterial microflora and the microflora within VPs among patients who had undergone total laryngectomy. We also explored the correlation between the predominant isolated etiological agent and the average lifespan of the VP. Our assessment also extended to evaluating the phonatory efficiency and the impact on quality of life due to valve fluid leakage, providing an overview of the challenges faced by these patients.

The study uncovered a wide variety of microorganisms in both oral rinses and VP brushings, with *Candida tropicalis* being the most frequently isolated yeast strain in VPs. *Staphylococcus aureus* was the most frequently isolated bacterial species in VPs, consistent with the existing literature. The absence of significant relationships between the predominant microorganism and the lifespan of the VPs suggests that the factors contributing to VPs’ dysfunction and material disintegration are complex and multifaceted, involving a combination of microbial interactions and other environmental factors.

The combination of the VHI and V-RQOL questionnaires has allowed us to understand the impact of phonatory prosthesis inefficiency. These tools have highlighted both the technical and personal aspects of voice disabilities, emphasizing the need for holistic patient care that addresses both the physical and emotional aspects of vocal health.

Nonetheless, it is important to acknowledge the limitations of our study, including the relatively small sample size of twenty patients and the specific study period of 2020 to 2023. Additionally, as the patients were brought to our attention solely when the vocal defects were noticeable, there was potential to overlook cases with minimal VP leakage.

This selection bias may have impacted the overall representation of VP-related issues, and the study’s exclusive focus on patients reporting problems of fluid leakage could limit the generalizability of our findings to the broader laryngectomy patient population. Therefore, further research with larger and more diverse cohorts will be necessary in the future to better understand the complex factors influencing the lifespan of VPs.

## Figures and Tables

**Table 1:** Main VP characteristics.

		h
Provox Vega	Atos Medical (Hörby, Sweden)	Equipped with a very internalized valve flap and an oval tracheal flange with a safety strap that interacts little with the lateral trachea walls.
Blom-Singer Advantage	Inhealt Technologies (Carpinteria, CA, USA)	Equipped with a silicone valve that contains silver oxide to prevent colonization and a titanium ring to prolong the material’s durability.
Blom-Singer Dual Valve	Inhealt Technologies (Carpinteria, CA, USA)	Equipped with two silver-oxide-treated silicone valves, to allow continued function in case of colonization around the primary area.

**Table 2:** Voice prosthesis models.

n	Voice Prosthesis Model
6	Provox Vega 22.5 Fr—10 mm
2	Provox Vega 22.5 Fr—6 mm
4	Provox Vega 20 Fr—10 mm
2	Provox Vega 20 Fr—8 mm
4	Blom-Singer Advantage 20 Fr—10 mm
2	Blom-Singer Dual Valve 20 Fr—8 mm

## Data Availability

The datasets presented in this article are not readily available because the data are part of an ongoing study.
